# Supplementary material for: Genome-wide association analysis identifies novel loci for chronotype in 100,420 individuals from the UK Biobank
Source: Nat Commun. 2016 Mar 9;7:10889. doi: 10.1038/ncomms10889 (PMC4786869; doi:10.1038/ncomms10889)
Supplement: Supplementary Information — Supplementary Figures 1-5, Supplementary Tables 1-13 and Supplementary Note 1 [file ncomms10889-s1.pdf]

Continuous Chronotype Extreme Chronotype

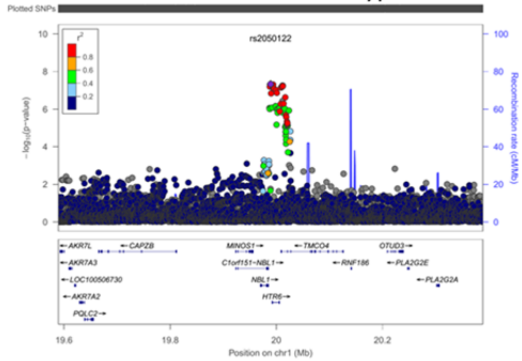

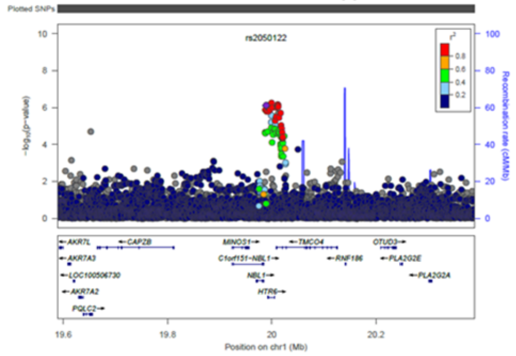

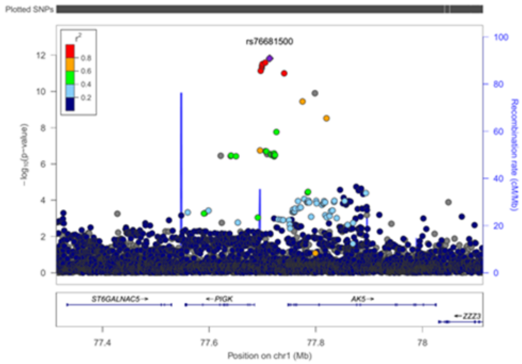

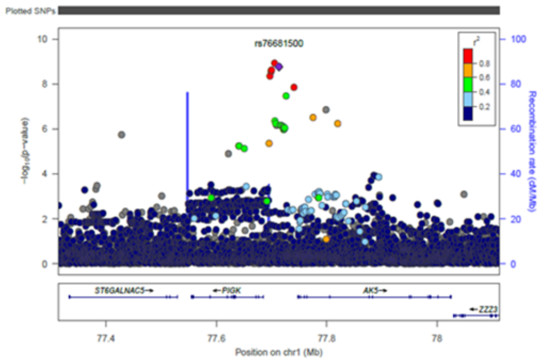

Continuous Chronotype Extreme Chronotype

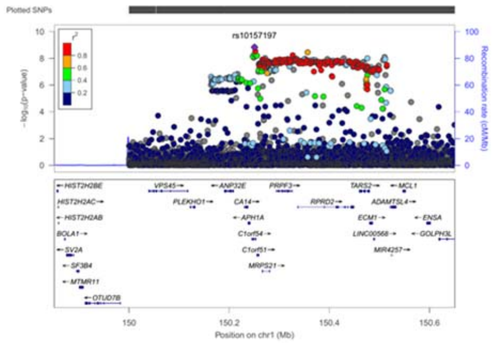

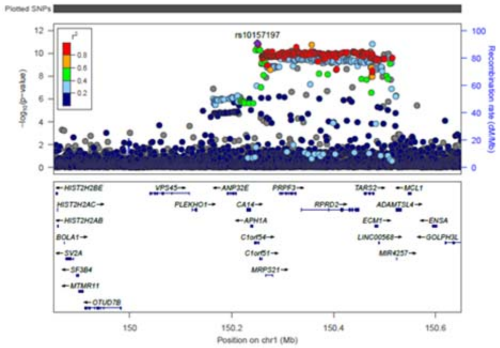

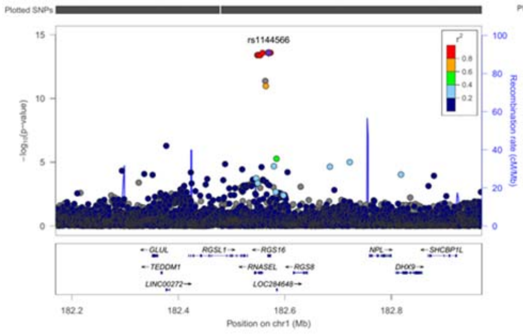

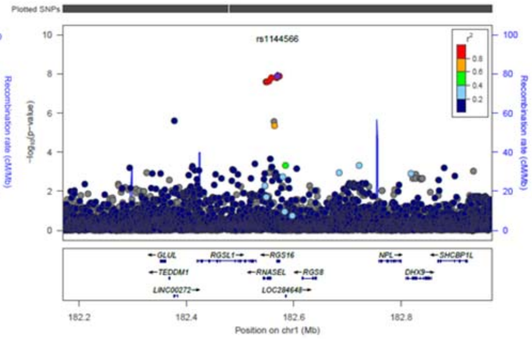

Continuous Chronotype	Extreme Chronotype

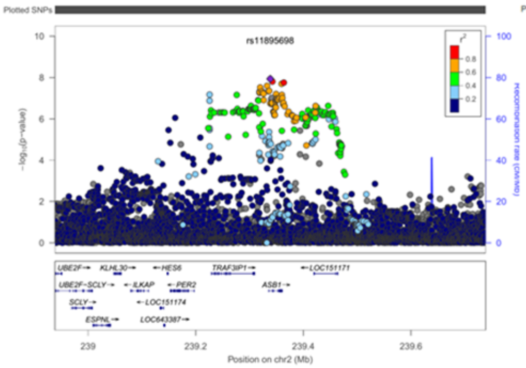

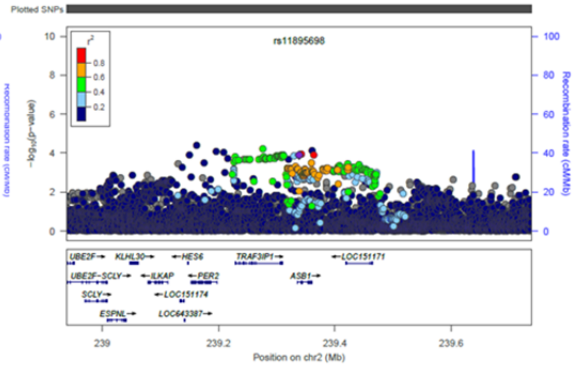

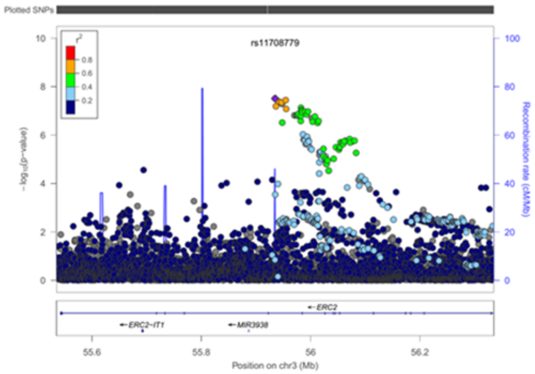

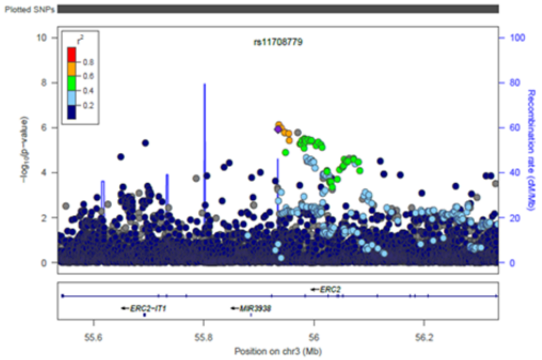

Continuous Chronotype	Extreme Chronotype

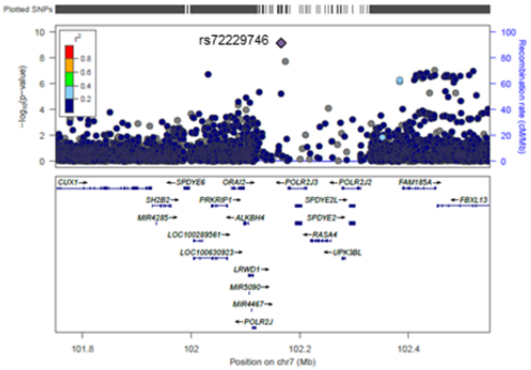

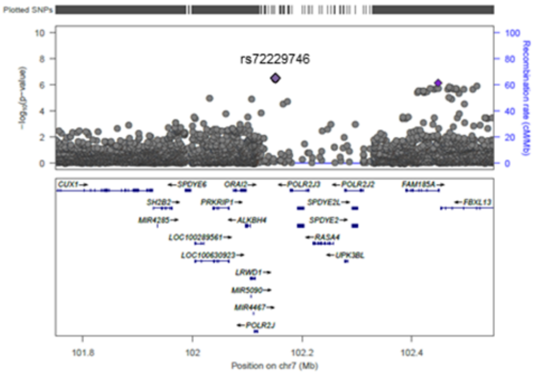

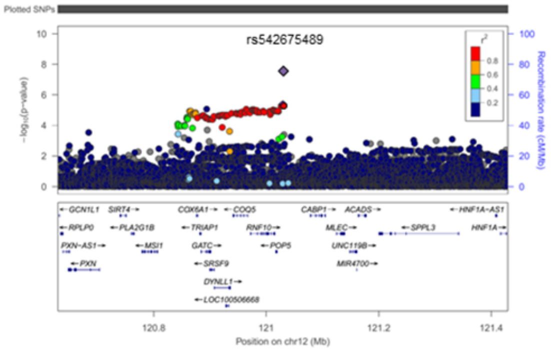

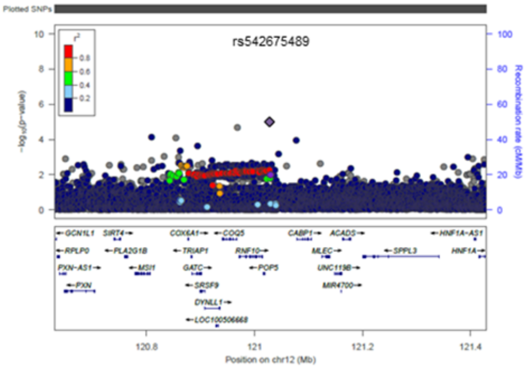

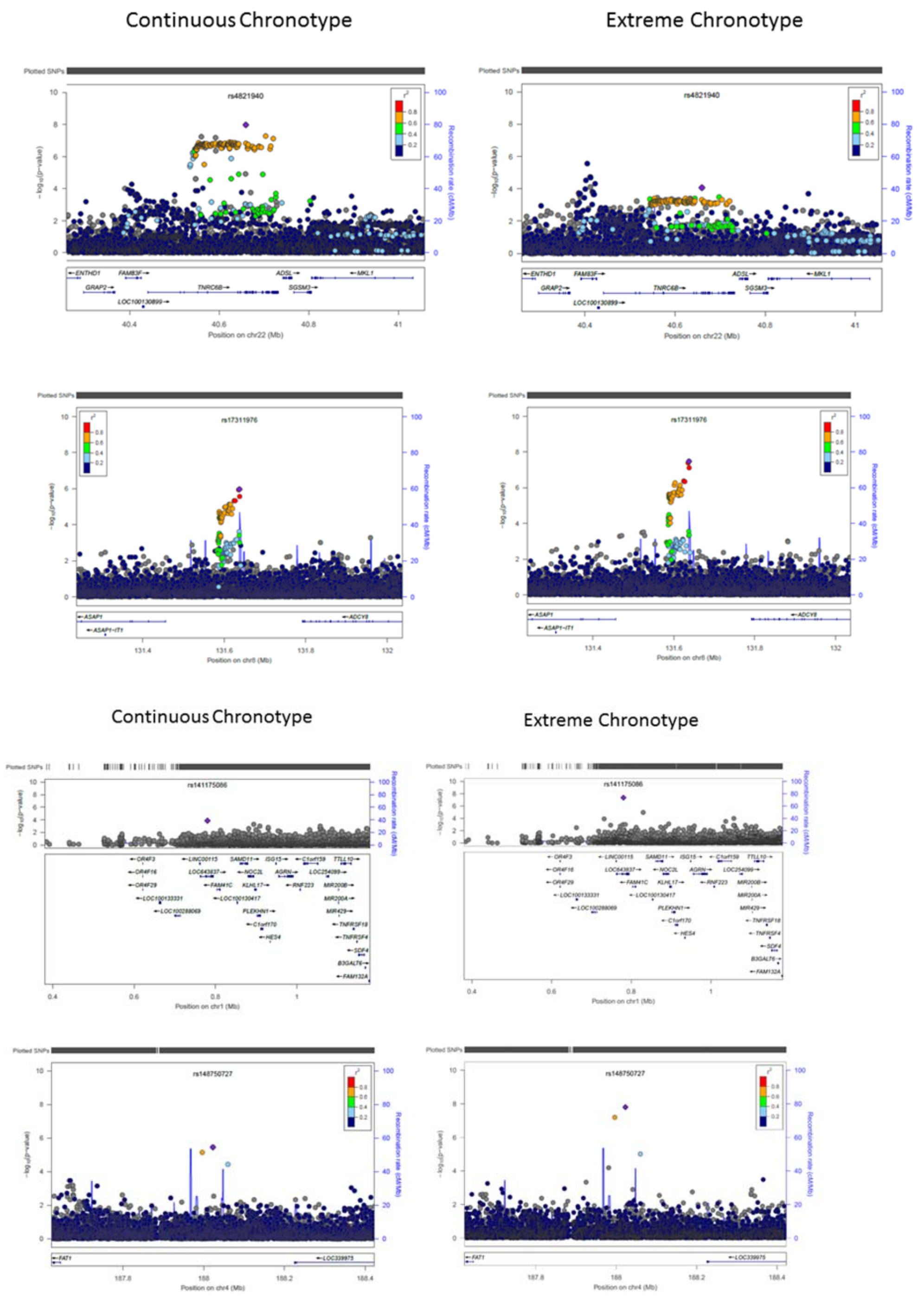

Supplementary Figure 1. Regional association plots for chronotype shown for both continuous and extreme chronotype. Genes within the region are shown in the lower panel. The blue line indicates the recombination rate. Filled circles show the log10 P value for each SNP, with the lead SNP shown in purple. Additional SNPs in the locus are colored according to correlation (r^2) with the lead SNP (estimated by LocusZoom based on the CEU HapMap haplotypes). *chr7 rs372229746 is not in the reference panel, therefore linkage data is unavailable for this SNP.

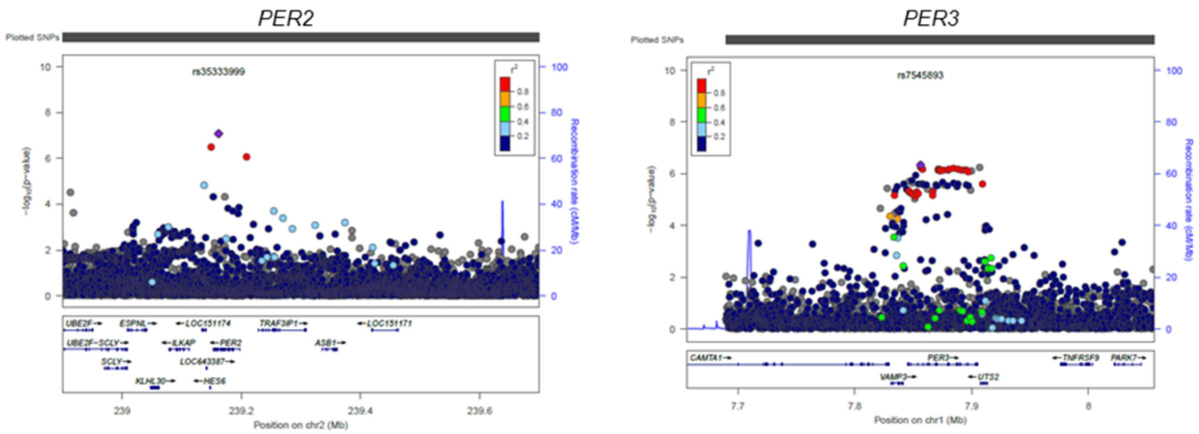

Supplementary Figure 2. Regional association plots for previously reported chronotype genes, *PER2* and *PER3*. A. A secondary signal is seen in *PER2* at a missense variant associated with continuous chronotype after conditioning on our lead SNP (rs11895698). B. A VNTR in *PER3* has been previously reported to associate with chronotype, we find a suggestive signal ~30kb upstream of the untyped VNTR region. Genes within the region are shown in the lower panel. The blue line indicates the recombination rate. Filled circles show the log10 P value for each SNP, with the lead SNP shown in purple. Additional SNPs in the locus are colored according to correlation (r^2) with the lead SNP (estimated by LocusZoom based on the CEU HapMap haplotypes).

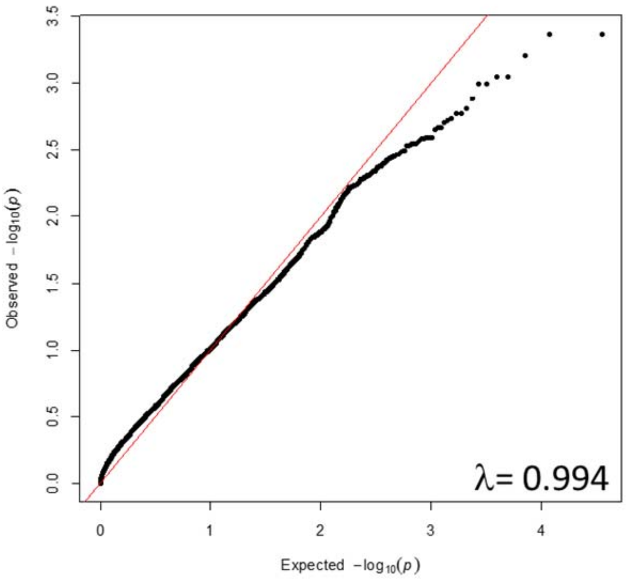

Supplementary Figure 3. Q-Q plot for inflation-adjusted gene-based genome-wide association analysis (VEGAS) of continuous chronotype.

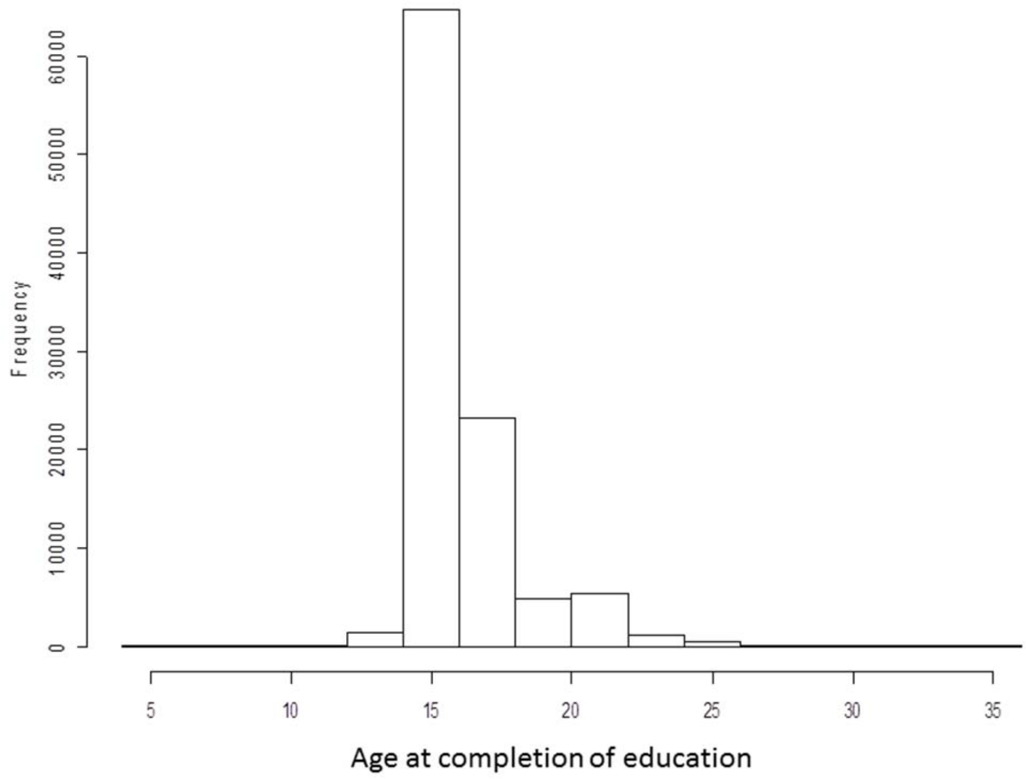

Supplementary Figure 4. Histogram of age at completion of education in the UKBiobank. The average age of completion is 16, representing completion of primary education in the UK, with a second minor peak at age 21, representing university level education.

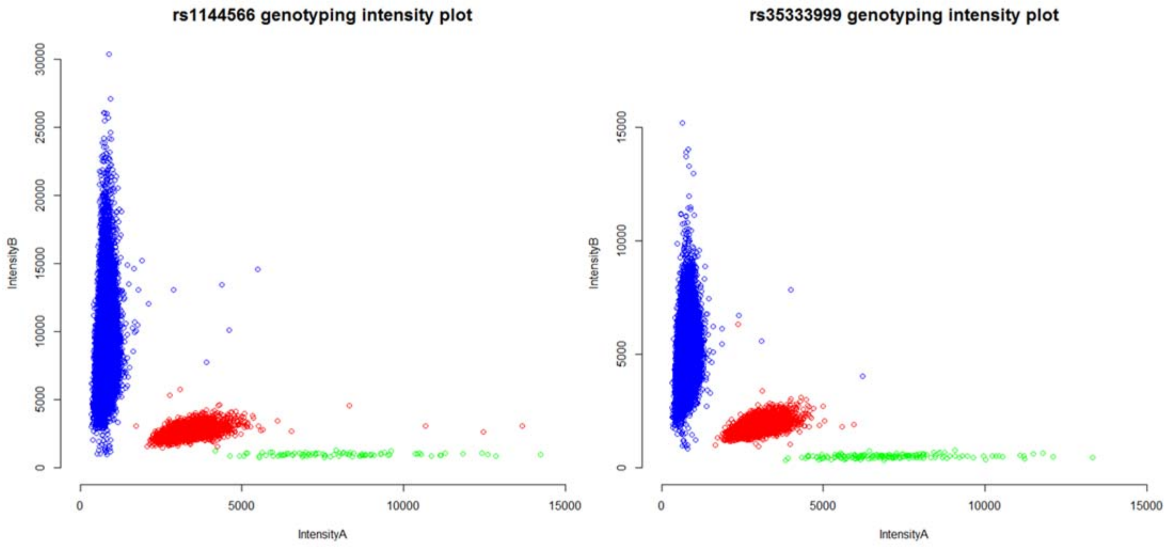

Supplementary Figure 5. Cluster plots for significant rare SNPs directly genoyped by the UK Biobank. Red, green and blue represent AA, AB and BB homozygotes, respectively.

Supplementary Table 1. Descriptive Characteristics of UKBiobank subjects of European ancestry used for GWAS (N=100,420).

**Characteristic**	**Mean ± Standard Deviation** **N (%)**	**Regression with Chronotype** Beta, p-value
Sex (M)	52,610 (47%)	0.028, $p=6.5x10^{-4}$
Age, y	57.68 (7.90)	-0.011, p<0.0001
Depression	4279 (4.3%)	0.0108, p<0.0001
Psychiatric Medication	1540 (1.5%)	0.084, p<0.0001
Chronotype		
Definite morning (1)	26,948 (27%)	-
More morning (2)	36,220 (36%)	-
More evening (3)	28,528 (28%)	-
Definite evening (4)	8,724 (9%)	-
Sleep duration, hrs	7.18 (1.08)	0.033, p<0.0001
Sleep disturbance		0.017, $p=3.7x10^{-5}$
Never/Rarely	23,989 (24%)	
Sometimes	47,711 (48%)	
Usually	28,677 (28%)	
Schizophrenia	86 (.1%)	0.311, p=0.0018
Age at education completion, yrs	16.41 (2.85)	0.0167, p<0.0001
BMI, kg/m2	29.23 (6.82)	1.068, p=0.0001

Beta reflects change in continuous chronotype 1-4, where 1 is "definite morning" and 4= "definite evening"). BMI=body mass index.

Supplementary Table 2. Secondary signals after conditioning on lead GWS SNP for chronotype at each locus.

Secondary Signal after Conditioning on Lead GWS SNP								
**Lead SNP**	**Chr:position**	**Secondary SNP**	**Chr:position**	**Nearest Gene**	**Alleles (E/A)**	**N**	**Beta (SE)**	***p-val***
rs141175086	1:780,397	rs564505457	1:1199763	*UBE2J2*	G/C	100,420	0.293 (0.079)	2.07E-04
rs2050122	1:19,989,205	rs538034991	1:19824482	*NBL1/ MINOS1*	G/C	100,420	0.442 (0.122)	2.90E-04
rs76681500	1:77,713,434	rs200652891	1:78048791	*ZZZ3*	CA/C	100,420	0.212 (0.057)	2.10E-04
rs10157197	1:150,250,636	rs181022810	1:150168490	*THEM4/CTMP*	C/A	100,420	0.207 (0.053)	9.68E-05
rs1144566	1:182,569,626	rs76843577	1:182605105	*TSEN15*	A/G	100,420	0.286 (0.070)	4.25E-05
**rs11895698**	**2:239,338,495**	**rs35333999**	**2:239161957**	***PER2***	**T/C**	100,420	**0.059 (0.011)**	**8.43E-08**
rs11708779	3:55,934,939	rs552069606	3:55867508	*ERC2*	C/A	100,420	0.103 (0.025)	4.13E-05
rs148750727	4:188,022,952	rs569386778	4:187657423	*F11-AS1*	T/C	100,420	0.161 (0.041)	8.49E-05
rs372229746	7:102,158,815	rs117530689	7:102030935	*POLR2J3/RASA4*	C/A	100,420	0.060 (0.013)	3.58E-06
rs17311976	8:131637337	rs569482456	8:131995157	*ADCY8*	C/T	100,420	0.718 (0.234)	1.00E-03
rs542675489	12:120,994,888	rs188978844	12:120684639	*PXN*	T/C	100,420	0.134 (0.034)	8.45E-05
rs4821940	22:40,659,573	rs3021269	22:40404823	*FAM83F*	T/C	100,420	0.339 (0.088)	1.17E-04

Conditional association results include a ±500kb window around the lead SNP. Chr=chromosome, E=effect allele, A=alternate allele, SE=standard error.

Supplementary Table 3. Genome-Wide significant associations of genetics variants with morningness in subjects of European ancestry from the 23andMe and UKBiobank cohorts.

					**23andMe* morning n=38,937 evening n=50,346**			**UKBiobank morning n=26,948 evening n=8,724**				**Meta-Analysis morning n=65,997 evening n=59,126**
**Gene**	**Marker**	**Chr**	**Position (37/hg19)**	**Alleles (E/A)**	**EAF**	**OR**	***P*-value**	**EAF**	**OR**	**95% CI**	***P*-value**	**P-value**
PER3	**rs11121022**	**1**	**7836659**	**C/A**	**0.42**	**1.07**	**2.0×10^{-8}**	**0.43**	**1.06**	**1.03-1.10**	**4.38×10^{-4}**	**2.96×10^{-8}**
AK5	**rs10493596**	**1**	**77726241**	**T/C**	**0.24**	**1.09**	**8.0×10^{-12}**	**0.23**	**1.13**	**1.08-1.17**	**2.05×10^{-8}**	**1.76×10^{-15}**
APH1A	**rs34714364**	**1**	**150234657**	**T/G**	**0.17**	**1.12**	**2.0×10^{-10}**	**0.17**	**1.12**	**1.07-1.18**	**1.76×10^{-6}**	**2.47×10^{-12}**
RGS16	**rs12736689**	**1**	**182549729**	**C/T**	**0.03**	**1.35**	**7.0×10^{-18}**	**0.03**	**1.34**	**1.20-1.49**	**3.62×10^{-8}**	**7.23×10^{-18}**
PLCL1	rs1595824**	2	198874006	T/C	0.49	1.08	1.2×10^{-10}	0.5	1.02	0.99-1.06	0.167	7.72×10^{-5}
PER2	**rs55694368**	**2**	**239317692**	**G/T**	**0.93**	**1.16**	**2.6×10^{-9}**	**0.89**	**1.08**	**1.02-1.14**	**5.15×10^{-3}**	**4.77×10^{-7}**
HCRTR2	**rs35833281**	**6**	**55021561**	**C/G**	**0.21**	**1.09**	**3.7×10^{-9}**	**0.24**	**1.06**	**1.02-1.11**	**1.9×10^{-3}**	**1.22×10^{-7}**
VIP	**rs9479402**	**6**	**153135339**	**C/T**	**0.01**	**1.45**	**3.9×10^{-11}**	**0.01**	**1.35**	**1.12-1.63**	**1.2×10^{-3}**	**1.13×10^{-8}**
DLX5	rs2948276	7	96457119	A/G	0.82	1.09	1.1×10^{-8}	0.83	1.05	1.01-1.10	0.014	3.74×10^{-6}
FBXL13	rs3972456	7	102436907	A/G	0.29	1.09	6.0×10^{-9}	0.34	1.05	1.02-1.09	0.01	1.79×10^{-6}
ALG10B	rs6582618	12	38726137	G/A	0.52	1.07	1.5×10^{-8}	0.54	1.03	1.00-1.07	0.039	2.18×10^{-5}
FBXL3	**rs9565309**	**13**	**77577027**	**T/C**	**0.97**	**1.19**	**3.5×10^{-8}**	**0.97**	**1.14**	**1.03-1.25**	**7.91×10^{-3}**	**2.34×10^{-6}**
RASD1	rs11545787	17	17398278	G/A	0.76	1.08	1.4×10^{-8}	0.75	1.04	1.00-1.08	0.084	7.97×10^{-5}
NOL4	rs12965577	18	31675680	A/G	0.66	1.06	2.1×10^{-8}	0.67	1.03	0.99-1.06	0.159	2.89×10^{-4}
TOX3	**rs12927162**	**16**	**52684916**	**A/G**	**0.74**	**1.1**	**1.6×10^{-12}**	**0.72**	**1.07**	**1.03-1.11**	**8.02×10^{-4}**	**1.92×10^{-9}**

Results are from logistic regression analysis adjusted for age,sex, principal components of ancestry and in the UKBiobank sample only, genotyping platform. *indicates publicly available data from a meeting abstract. ** proxy rs11683222 C/G

Supplementary Table 4. Previously reported variants for chronotype-related traits do not associate with chronotype in the UKBiobank.

			Continuous Chronotype			Extreme Chronotype				
**SNP**	**Chr:position**	**Alleles (E/A)**	**N**	**OR (95% CI)**	***p-val***	**N**	**OR (95% CI)**	***p-val***	**Citation**	**Phenotype**
rs1801260	4:56301369	A/G	100,420	1.01 [1.00-1.02]	1.23E-01	35,672	1.03 [0.99-1.07]	1.82E-01	Katzenberg D. et al. Sleep 1998	Evening preference
rs324981	7:34818113	A/T	100,420	1.01 [1.00-1.02]	7.75E-02	35,672	1.03 [1.00-1.07]	6.76E-02	Gottlieb DJ. et al. BMC Med Genet. 2007.	Bedtime
rs949175	11:97737007	T/G	100,420	1.05 [1.05-1.05]	1.74E-01	35,673	1.02 [0.98-1.06]	2.78E-01		
rs2288292	11:12495699	T/C	100,420	1.00 [0.99-1.01]	8.16E-01	35,672	1.02 [0.97-1.07]	4.92E-01		
rs10507551	13:47761883	G/A	100,420	1.01 [1.00-1.01]	2.47E-01	35,672	1.02 [0.98-1.05]	3.81E-01		
rs10483871	14:76071281	C/G	100,421	1.00 [0.99-1.01]	8.68E-01	35,672	1.01 [0.96-1.06]	7.89E-01		
rs2985334	1:29345027	G/T	100,420	1.00 [0.99-1.01]	4.10E-01	35,673	1.04 [1.00-1.08]	5.17E-02		
rs2525724	7:120609943	A/G	100,419	1.01 [1.00-1.02]	1.40E-01	35,671	1.03 [0.99-1.07]	1.57E-01		
rs1725021	7:136893320	T/C	100,420	1.00 [0.99-1.01]	7.19E-01	35,671	1.01 [0.98-1.05]	5.09E-01		
rs932650	10:115347359	C/T	100,420	0.99 [0.98-1.00]	8.81E-02	35,672	1.05 [1.01-1.09]	1.25E-02		
rs1940013	11:132281651	T/C	100,420	1.00 [0.99-1.01]	5.55E-01	35,673	1.00 [0.97-1.04]	8.97E-01		
rs28936679	17:74465813	A/G	100,417	2.87 [0.99-8.33]	5.21E-02	-	-	-	Hohjoh H. et al. Neurogenetics 2003.	DSPS

Supplementary Table 5. Association of GWS Chronotype Loci with Morning or Evening Chronotype.

**Phenotype**	**SNP**	**Chr:position**	**Alleles (E/A)**	**EAF**	**N**	**OR (95% CI)**	***p-val***
Morning vs. middle	rs141175086	1:780,397	T/C	0.002	92,058	1.30 [0.97-1.75]	8.47E-02
	rs2050122	1:19,989,205	T/C	0.196	92,058	1.04 [1.01-1.07]	2.29E-03
	rs76681500	1:77,713,434	A/G	0.159	92,058	1.06 [1.03-1.09]	1.24E-05
	rs10157197	1:150,250,636	G/A	0.602	92,058	1.02 [1.00-1.04]	1.26E-01
	rs1144566	1:182,569,626	T/C	0.030	92,058	1.16 [1.09-1.23]	4.58E-07
	rs11895698	2:239,338,495	C/T	0.857	92,058	1.04 [1.01-1.07]	5.47E-03
	rs11708779	3:55,934,939	A/G	0.352	92,058	1.05 [1.03-1.07]	1.02E-05
	rs148750727	4:188,022,952	G/T	0.005	92,058	1.18 [1.02-1.36]	2.58E-02
	rs372229746	7:102,158,815	G/A	0.552	92,058	1.05 [1.03-1.08]	3.40E-05
	rs17311976	8:131,637,337	T/C	0.810	92,058	1.04 [1.01-1.07]	2.74E-03
	rs542675489	12:120,994,888	CA/C	0.404	92,057	1.05 [1.03-1.07]	7.05E-06
	rs4821940	22:40,659,573	T/C	0.448	92,059	1.04 [1.02-1.07]	2.18E-05
Evening vs. middle	rs141175086	1:780,397	C/T	0.998	73,778	4.46 [2.02-9.88]	1.65E-05
	rs2050122	1:19,989,205	C/T	0.804	73,778	1.08 [1.03-1.12]	4.12E-04
	rs76681500	1:77,713,434	G/A	0.841	73,778	1.10 [1.05-1.15]	3.07E-05
	**rs10157197**	**1:150,250,636**	A/G	0.398	**73,778**	**1.11 [1.07-1.15]**	**2.33E-10**
	rs1144566	1:182,569,626	C/T	0.970	73,778	1.16 [1.05-1.28]	3.58E-03
	rs11895698	2:239,338,495	T/C	0.143	73,778	1.05 [1.01-1.10]	1.53E-02
	rs11708779	3:55,934,939	G/A	0.648	73,778	1.04 [1.01-1.08]	2.01E-02
	rs148750727	4:188,022,952	T/G	0.995	73,778	1.88 [1.38-2.56]	1.40E-05
	rs372229746	7:102,158,815	A/G	0.448	73,778	1.06 [1.02-1.10]	3.24E-03
	rs17311976	8:131,637,337	C/T	0.193	73,778	1.08 [1.04-1.13]	9.49E-05
	rs542675489	12:120,994,888	C/CA	0.596	73,778	1.03 [1.00-1.07]	7.66E-02
	rs4821940	22:40,659,573	C/T	0.552	73,779	1.03 [0.99-1.06]	1.02E-01

GWS loci were tested for association with either definite morning or definite evening chronotype compared to the mild middle chronotype responses. Effect allele is reported as the morningness or eveningness allele for each association.

Supplementary Table 6. Sex does not significantly modify the effect of genetic variants on Chronotype.

					Female (N=54,548)			Male (N=45,872)		
**SNP**	**Chr**	**Position**	**Alleles (E/A)**	**interaction *p-val***	**EAF**	**Beta (SE)**	***p-val***	**EAF**	**Beta (SE)**	***p-val***
rs10157197	1	150250636	A/**G**	0.195	0.398	0.034 (0.006)	6.69E-08	0.399	0.020 (0.007)	2.45E-03
rs1144566	1	182569626	**C**/T	0.269	0.970	0.113 (0.018)	1.55E-10	0.970	0.082 (0.019)	1.82E-05
rs2050122	1	19989205	C/**T**	0.628	0.804	0.034 (0.008)	8.38E-06	0.805	0.027 (0.008)	1.14E-03
rs76681500	1	77713434	**G**/A	0.555	0.841	0.047 (0.008)	1.93E-08	0.841	0.039 (0.009)	1.50E-05
rs141175086	1	780397	**C**/T	0.466	0.998	0.217 (0.093)	1.96E-02	0.998	0.331 (0.106)	1.85E-03
rs11895698	2	239338495	T/**C**	0.803	0.144	0.035 (0.008)	3.05E-05	0.143	0.035 (0.009)	9.71E-05
rs11708779	3	55934939	**G**/A	0.590	0.649	0.028 (0.006)	9.33E-06	0.647	0.023 (0.007)	8.04E-04
rs148750727	4	188022952	T/**G**	0.339	0.995	0.132 (0.045)	3.37E-03	0.995	0.180 (0.049)	2.71E-04
rs372229746	7	102158815	A/G	0.538	0.448	0.037 (0.007)	5.05E-07	0.448	0.030 (0.008)	2.42E-04
rs17311976	8	131637337	C/T	0.657	0.191	0.030 (0.008)	1.16E-04	0.190	0.025 (0.008)	2.75E-03
rs542675489	12	120994888	C/CA	0.424	0.597	0.030 (0.007)	3.64E-06	0.594	0.022 (0.007)	1.81E-03
rs4821940	22	40659573	C/**T**	0.478	0.552	0.023 (0.006)	1.71E-04	0.553	0.029 (0.007)	1.18E-05

E=effect allele, A=alternative allele. Ancestral allele is indicated in bold. EAF=effect allele frequency. Interaction p-val is from regression model with interaction term SEX x SNP using continuous chronotype. Beta values are from regression tests stratified on sex.

Supplementary Table 7. Epistasis between index SNPs from GWS loci for chronotype ($p<0.05$)

CHR1	SNP1	CHR2	SNP2	BETA_INT	P
7	rs3972456	12	rs6582618	-0.018	0.0023
4	rs148750727	16	rs12927162	-0.147	0.0056
1	rs141175086	1	rs11121022	0.374	0.0113
1	rs10157197	8	rs17311976	-0.019	0.0123
18	rs12965577	22	rs4821940	0.015	0.0148
1	rs10493596	18	rs12965577	0.016	0.0255
6	rs35833281	7	rs3972456	0.016	0.0286
1	rs10157197	2	rs11895698	0.019	0.0297
1	rs1144566	7	rs372229746	-0.060	0.0304
1	rs2050122	1	rs10157197	0.016	0.0415
1	rs141175086	12	rs6582618	0.255	0.0431
1	rs12736689	7	rs372229746	-0.056	0.0450
1	rs34714364	1	rs12736689	0.047	0.0457
2	rs11895698	7	rs372229746	-0.027	0.0476
1	rs76681500	7	rs372229746	-0.026	0.0495
7	rs3972456	8	rs17311976	0.016	0.0495

Pair-wise interaction tests were conducted in PLINK and included SNPs from the UKBiobank and 23andME. No SNPs passed the multiple testing Pvalue threshold.

Supplementary Table 8. Sensitivity analyses adjusting for factors known to associate with chronotype.

SNP	Chr	Position	Effect Allele	Alt Allele	Additional Adjustments	Beta	SE	*P*
rs2050122	1	19989205	C	T	-	0.031	0.006	4.61E-08
					Insomnia	0.031	0.006	4.60E-08
					Psychiatric Illness	0.029	0.006	2.93E-07
					Sleep Disorder	0.031	0.006	4.60E-08
					Sleep Disorder+Psychiatric Illnes	0.029	0.006	2.92E-07
					Sleep Duration	0.031	0.006	4.33E-08
rs76681500	1	77713434	A	G	-	-0.043	0.006	1.50E-12
					Insomnia	-0.043	0.006	1.94E-12
					Psychiatric Illness	-0.044	0.006	1.39E-12
					Sleep Disorder	-0.043	0.006	1.53E-12
					Sleep Disorder+Psychiatric Illnes	-0.044	0.006	1.41E-12
					Sleep Duration	-0.043	0.006	1.52E-12
rs10157197	1	150250636	A	G	-	0.028	0.005	1.48E-09
					Insomnia	0.027	0.005	2.46E-09
					Psychiatric Illness	0.028	0.005	1.44E-09
					Sleep Disorder	0.028	0.005	1.51E-09
					Sleep Disorder+Psychiatric Illnes	0.028	0.005	1.45E-09
					Sleep Duration	0.028	0.005	1.32E-09
rs11895698	2	239338495	T	C	-	0.035	0.006	1.15E-08
					Insomnia	0.035	0.006	1.17E-08
					Psychiatric Illness	0.037	0.006	3.22E-09
					Sleep Disorder	0.035	0.006	1.18E-08
					Sleep Disorder+Psychiatric Illnes	0.037	0.006	3.26E-09
					Sleep Duration	0.035	0.006	1.06E-08
rs11708779	3	55934939	A	G	-	-0.026	0.005	3.08E-08
					Insomnia	-0.026	0.005	2.53E-08
					Psychiatric Illness	-0.025	0.005	9.70E-08
					Sleep Disorder	-0.026	0.005	3.13E-08
					Sleep Disorder+Psychiatric Illnes	-0.025	0.005	9.75E-08
					Sleep Duration	-0.026	0.005	3.03E-08
rs37222974	7	102158815	A	G	-	0.034	0.005	5.18E-10
					Insomnia	0.034	0.005	5.42E-10
					Psychiatric Illness	0.035	0.006	3.70E-10
					Sleep Disorder	0.034	0.005	5.28E-10
					Sleep Disorder+Psychiatric Illnes	0.035	0.006	3.73E-10
					Sleep Duration	0.034	0.005	5.55E-10
rs4821940	22	40659573	C	T	-	0.026	0.004	1.05E-08
					Insomnia	0.026	0.004	1.05E-08
					Psychiatric Illness	0.025	0.005	5.32E-08
					Sleep Disorder	0.026	0.004	1.08E-08
					Sleep Disorder+Psychiatric Illnes	0.025	0.005	5.36E-08
					Sleep Duration	0.026	0.004	7.70E-09

Supplementary Table 9. Gene sets enriched in candidate chronotype genes from significant loci.

Database	GeneSet	Genes	Enrichment	$P_{Adjusted}$
Disease	Mental Disorders	*ADSL,PER3,APH1A,FAT1,HCRTR2, HTR6,PER2*	8.23	0.001
	Seasonal affective disorder	*PER2,PER3*	94.78	0.003
	Depression	*PER3,VIP,HTR6,PER2*	14.42	0.003
	Fatigue Syndrome, Chronic	*RNASEL,HTR6*	47.39	0.005
	Depressive Disorder	*PER3,HTR6,PER2*	17.77	0.005
	Sleep Disorders	*PER3,HCRTR2,PER2*	17.93	0.005
	Paranoid psychosis NOS	*HTR6,PER2*	57.69	0.005
	Mood Disorders	*PER3,FAT1,HTR6,PER2*	10.29	0.005
	Psychotic Disorders	*PER3,HTR6,PER2*	15.43	0.005
	Bipolar Disorder	*PER3,FAT1,VIP,PER2*	7.72	0.009
	Eating Disorders	*VIP,HCRTR2,HTR6*	11.57	0.009
	Fatigue	*RNASEL,PER3*	28.23	0.009
	Nelson syndrome	*RASA4,ORAI2,SPDYE2L,SHFM1, FBXL13*	4.93	0.013
	Glaucoma	*ADAMTSL4,SRSF9*	14.27	0.026
KEGG pathway	Spliceosome	*PRPF3, SRSF9*	10.45	0.020
	Alzheimer's disease	*APH1A, COX6A1*	7.95	0.030
	Neuroactive ligand-receptor interaction	*HCRTR2, HTR6*	4.88	0.064
Wikipathways pathway	Diurnally regulated genes with circadian orthologs	*ERC2, PER2*	27.65	0.014

Enrichment was tested using WebGestalt from a list of genes in the region in LD with lead chronotype loci (r^2>0.2).

Supplementary Table 10a. Genetic correlation between chronotype and 19 traits and partitioned heritability.

**Trait 1**	**Trait 2**	**Correlation rg (SE)**	***p-val***
**Chronotype**	**Years of Education**	**0.161 (0.041)**	**8.96E-05**
	**Schizophrenia**	**0.112 (0.034)**	**1.10E-03**
	**BMI 2015**	**-0.085 (0.028)**	**2.50E-03**
	Former/Current Smoker	0.206 (0.072)	4.00E-03
	Autism Spectrum	0.161 (0.065)	1.37E-02
	Depression	0.185 (0.076)	1.49E-02
	Coronary Artery Disease	-0.124 (0.054)	2.05E-02
	Waist-Hip Ratio	-0.063 (0.033)	5.29E-02
	Infant Head Circumference	-0.136 (0.073)	6.37E-02
	Total Cholesterol	0.072 (0.041)	7.85E-02
	Anorexia	0.054 (0.046)	2.36E-01
	Bipolar	0.063 (0.058)	2.71E-01
	Neck BMD	-0.044 (0.041)	2.82E-01
	Rheumatoid Arthritis	-0.049 (0.055)	3.77E-01
	Crohn's Disease	0.031 (0.04)	4.32E-01
	Alzheimer's	-0.053 (0.082)	5.18E-01
	ADHD	0.061 (0.099)	5.38E-01
	T2D	-0.009 (0.054)	8.71E-01
	Height	-0.003 (0.023)	8.93E-01

After Bonferroni correction, p-value cut-off is 0.0026. Correlation and partitioned heritability is measured using LD Score Regression (LDSC). Positive correlation represents a correlation with eveningness and negative correlation represents a correlation with morningness. GWAS data for all 19 traits is publicly available.

Supplementary Table 10b. Genetic correlation between chronotype and 19 traits and partitioned heritability: heritability partitioned across tissue type.

Partitioned Heritability, Tissue

Cell Type Group	Proportion of SNPs	Prop. h2	Prop. h2 SE	Enrichment	Enrichment SE	Enrichment p-val
Connective/Bone	0.115	0.140	0.042	1.217	0.364	5.51E-01
Gastrointestinal	0.168	0.210	0.056	1.255	0.334	4.46E-01
Cardiovascular	0.111	0.231	0.058	2.080	0.525	3.98E-02
Liver	0.072	0.156	0.038	2.158	0.532	2.94E-02
Kidney	0.043	0.121	0.036	2.834	0.837	2.84E-02
SkeletalMuscle	0.104	0.229	0.048	2.204	0.460	8.81E-03
Immune	0.233	0.366	0.069	1.567	0.298	5.70E-02
**CNS**	**0.149**	**0.391**	**0.051**	**2.630**	**0.342**	**1.91E-06**
**Adrenal/Pancreas**	**0.094**	**0.340**	**0.050**	**3.631**	**0.537**	**9.60E-07**

SE=standard error. h2=heritability. Prop=proportion.

Supplementary Table 10c. Genetic correlation between chronotype and 19 traits and partitioned heritability. Heritability partitioned across functional class.

Functional Class	Proportion of SNPs	Prop. h2	Prop. h2 SE	Enrichment	Enrichment SE	Enrichment p-val
29 mammals conserved, extended	0.333	0.641	0.072	1.929	0.217	1.83E-05
H3K4me1, extended	0.609	0.807	0.059	1.324	0.096	7.42E-04
TSS, extended	0.035	0.149	0.038	4.273	1.084	2.53E-03
Coding	0.015	0.104	0.033	7.086	2.256	6.97E-03
H3K9ac_peaks	0.039	0.248	0.078	6.389	2.007	7.25E-03
H3K9ac, extended	0.231	0.411	0.068	1.781	0.295	8.15E-03
TSS	0.018	0.139	0.046	7.627	2.535	8.94E-03
3-PrimeUTR	0.011	0.075	0.026	6.777	2.358	1.43E-02

H3K4me1_peaks	0.171	0.463	0.129	2.705	0.755	2.39E-02
Super Enhancer, extended	0.172	0.236	0.030	1.373	0.176	3.38E-02
H3K9ac	0.126	0.289	0.082	2.293	0.647	4.57E-02
H3K4me3	0.133	0.275	0.074	2.059	0.556	5.67E-02
DHS, extended	0.499	0.711	0.117	1.426	0.235	7.02E-02
Repressed, extended	0.719	0.656	0.043	0.912	0.060	1.40E-01
FetalDHS, extended	0.285	0.431	0.107	1.512	0.374	1.71E-01
FetalDHS	0.085	0.237	0.114	2.791	1.344	1.83E-01
H3K27ac	0.269	0.373	0.083	1.383	0.308	2.15E-01
Intron, extended	0.397	0.440	0.035	1.108	0.087	2.15E-01
H3K4me3_peaks	0.042	0.132	0.074	3.148	1.775	2.26E-01
3-PrimeUTR, extended	0.027	0.064	0.031	2.370	1.140	2.30E-01
CTCF, extended	0.071	0.148	0.067	2.086	0.936	2.46E-01
H3K27ac, extended	0.336	0.414	0.070	1.232	0.210	2.68E-01
DGF, extended	0.542	0.644	0.099	1.189	0.184	3.03E-01
Promoter Flanking, extended	0.033	0.078	0.045	2.344	1.337	3.15E-01
Fantom5 Enhancer	0.004	-0.023	0.028	-5.300	6.410	3.26E-01
Promoter, extended	0.039	0.067	0.030	1.745	0.770	3.33E-01
Promoter Flanking	0.008	0.037	0.032	4.358	3.785	3.75E-01
Transcribed, extended	0.763	0.710	0.060	0.930	0.079	3.75E-01
H3K4me1	0.427	0.521	0.112	1.222	0.262	3.97E-01
Repressed	0.461	0.377	0.117	0.817	0.253	4.69E-01
Coding, extended	0.065	0.091	0.038	1.414	0.596	4.87E-01
CTCF	0.024	-0.015	0.065	-0.640	2.718	5.46E-01
Weak Enhancer	0.021	0.052	0.052	2.486	2.479	5.49E-01
5-PrimeUTR, extended	0.028	0.047	0.032	1.673	1.144	5.56E-01
Super Enhancer	0.168	0.186	0.031	1.106	0.182	5.61E-01
Transcribed	0.345	0.402	0.099	1.163	0.287	5.70E-01
Enhancer	0.063	0.029	0.069	0.455	1.095	6.18E-01
DHS peaks	0.112	0.047	0.138	0.424	1.235	6.41E-01
DGF	0.138	0.193	0.137	1.406	0.994	6.83E-01

5-PrimeUTR	0.005	0.013	0.018	2.339	3.308	6.86E-01
Fantom5 Enhancer, extended	0.019	0.032	0.034	1.682	1.791	7.03E-01
Promoter	0.031	0.017	0.041	0.552	1.325	7.35E-01
TFBS, extended	0.343	0.374	0.102	1.089	0.297	7.64E-01
Weak Enhancer, extended	0.089	0.073	0.055	0.818	0.620	7.69E-01
TFBS	0.132	0.115	0.116	0.866	0.878	8.79E-01
H3K4me3, extended	0.255	0.245	0.077	0.959	0.301	8.91E-01
Intron	0.387	0.392	0.037	1.012	0.094	8.99E-01
DHS	0.168	0.183	0.150	1.091	0.894	9.19E-01
Enhancer, extended	0.154	0.147	0.068	0.957	0.443	9.22E-01

SE=standard error. h2=heritability.
Prop=proportion.

Supplementary Table 11. Gene based association results for continuous chronotype using VEGAS.

Chr	*Gene*	nSNPs	nSims	Start	Stop	Pvalue	Genomic Control Pvalue	Best-SNP	SNP-pvalue
1	*ANP32E*	87	1000000	148457478	148475084	0	0	rs10157197	1.48E-09
1	*APH1A*	93	1000000	148504422	148508156	0	0	rs10157197	1.48E-09
1	*C1orf51*	97	1000000	148521852	148526125	0	0	rs10157197	1.48E-09
1	*C1orf54*	100	1000000	148511821	148519951	0	0	rs10157197	1.48E-09
1	*CA14*	97	1000000	148496841	148504102	0	0	rs10157197	1.48E-09
1	*MRPS21*	100	1000000	148532892	148547443	0	0	rs10157197	1.48E-09
1	*PRPF3*	124	1000000	148560551	148592328	0	0	rs10157197	1.48E-09
22	*TNRC6B*	308	1000000	38770766	39061758	0	0	rs4821940	1.05E-08
1	*KIAA0460*	188	1000000	148603613	148715665	0	0	rs834225	1.21E-08
1	*TARS2*	99	1000000	148726543	148746373	0	0	rs1566225	1.47E-08
1	*ECM1*	84	1000000	148747206	148752660	0	0	rs7513182	1.89E-08
1	*ADAMTSL4*	64	1000000	148788521	148800036	0	0	rs4971010	3.09E-08
1	*HTR6*	129	1000000	19864366	19878642	0	0	rs4912144	5.56E-08
1	*NBL1*	136	1000000	19842312	19857532	0	0	rs4912144	5.56E-08
9	*PAX5*	524	1000000	36828530	37024476	0	0	rs17485691	3.05E-07
12	*SMARCC2*	84	1000000	54843393	54869544	0	0	rs17118313	4.61E-04
12	*OBFC2B*	60	1000000	54904773	54909787	0	0	rs901073	5.57E-04
12	*RNF41*	66	1000000	54884552	54902002	0	0	rs901073	5.57E-04
21	*BAGE*	13	1000000	10079666	10120808	0	0	rs364756	3.46E-03
1	*SEC22B*	75	1000000	143807763	143828279	0	0	rs813452	1.69E-02
1	*TMCO4*	249	1000000	19881292	19998997	1.00E-06	5.52E-04	rs4912144	5.56E-08
13	*PCDH20*	190	1000000	60881819	60887656	1.00E-06	5.52E-04	rs17254585	9.18E-07
12	*SLC39A5*	61	1000000	54910893	54917896	2.00E-06	7.89E-04	rs901073	5.57E-04
12	*MYL6B*	67	1000000	54832601	54838038	4.00E-06	1.13E-03	rs773121	3.77E-04
12	*MYL6*	74	1000000	54838366	54841633	4.00E-06	1.13E-03	rs17118313	4.61E-04
12	*RIMBP2*	786	1000000	129446633	129568363	5.00E-06	1.27E-03	rs12810798	4.71E-04
1	*NOTCH2NL*	8	1000000	143920467	143997269	5.00E-06	1.27E-03	rs4068074	1.72E-02
2	*ASB1*	110	1000000	239000364	239025630	8.00E-06	1.62E-03	rs3769124	6.24E-08
12	*FZD10*	454	1000000	129212984	129216238	1.10E-05	1.91E-03	rs10848031	2.27E-03
6	*HLA-DPB1*	504	1000000	33151737	33162954	1.30E-05	2.08E-03	rs3117027	4.46E-06
12	*TRIAP1*	70	1000000	119366146	119368598	1.30E-05	2.08E-03	rs7957424	8.46E-06
1	*RGS16*	126	1000000	180834380	180840171	1.50E-05	2.24E-03	rs1144566	2.62E-14
14	*WDR25*	196	1000000	99912702	100066393	1.60E-05	2.31E-03	rs10148448	7.69E-05
12	*ALG10B*	165	1000000	36996823	37009795	1.70E-05	2.39E-03	rs6582630	1.76E-05
14	*ENTPD5*	145	1000000	73502933	73552386	2.10E-05	2.66E-03	rs17782281	1.15E-05
9	*IER5L*	299	1000000	130977651	130980361	2.10E-05	2.66E-03	rs9696811	1.08E-04
1	*KCNT2*	467	1000000	194461535	194844122	2.20E-05	2.73E-03	rs10801536	8.92E-06
18	*SEC11C*	245	1000000	54958104	54977043	2.80E-05	3.09E-03	rs9961653	3.38E-06
12	*COX6A1*	64	1000000	119360286	119362912	2.80E-05	3.09E-03	rs7957424	8.46E-06
12	*FAM62A*	74	1000000	54808320	54824721	2.80E-05	3.09E-03	rs773123	2.26E-04
7	*SLC26A5*	176	1000000	102780412	102873834	2.90E-05	3.15E-03	rs2190422	1.01E-06
12	*POP5*	87	1000000	119501230	119503584	2.90E-05	3.15E-03	rs7297861	5.30E-06
12	*RNF10*	113	1000000	119456514	119499780	3.10E-05	3.26E-03	rs7297861	5.30E-06
17	*PSME3*	46	1000000	38238948	38249303	3.30E-05	3.37E-03	rs9913866	3.66E-02
7	*TES*	210	1000000	115637816	115686073	3.40E-05	3.42E-03	rs6949241	7.70E-07
6	*GFRAL*	289	1000000	55300225	55375250	3.40E-05	3.42E-03	rs2653349	9.37E-07
12	*ANKRD52*	70	1000000	54917857	54938410	3.40E-05	3.42E-03	rs901073	5.57E-04
14	*C14orf45*	130	1000000	73555811	73602548	3.50E-05	3.47E-03	rs17782344	2.16E-05
9	*ZCCHC7*	330	1000000	37110468	37348145	3.60E-05	3.53E-03	rs17407627	3.37E-07
6	*HCRTR2*	309	1000000	55147029	55255377	3.60E-05	3.53E-03	rs2653344	6.01E-07
1	*BTF3L4*	110	1000000	52294560	52326650	4.30E-05	3.87E-03	rs12047841	5.19E-03
10	*ANXA8L1-1*	32	1000000	46577994	46594046	4.30E-05	3.87E-03	rs7921328	6.14E-02
12	*COQ5*	95	1000000	119425464	119451347	4.40E-05	3.91E-03	rs7957424	8.46E-06
1	*PIGK*	255	1000000	77327254	77457720	4.50E-05	3.96E-03	rs12040629	2.57E-12
7	*TBRG4*	154	1000000	45106223	45117842	4.80E-05	4.10E-03	rs7777835	3.47E-06
2	*ERMN*	107	1000000	157883370	157892392	4.80E-05	4.10E-03	rs6437054	2.26E-05
9	*FAM73B*	223	1000000	130839073	130874172	4.90E-05	4.14E-03	rs3118639	7.07E-05
1	*INADL*	572	1000000	61980736	62402179	5.10E-05	4.23E-03	rs2476194	4.39E-05

2	*C2orf34*	588	1000000	44442546	44853233	5.20E-05	4.27E-03	rs698820	5.88E-06
19	*SIGLEC11*	161	1000000	55144061	55156241	5.30E-05	4.31E-03	rs1290754	6.37E-04
10	*ANXA8-1*	32	1000000	46577989	46594128	5.30E-05	4.31E-03	rs7921328	6.14E-02
6	*HLA-DPA1*	471	1000000	33140771	33149356	5.40E-05	4.36E-03	rs3117027	4.46E-06
12	*SFRS9*	75	1000000	119383853	119391941	5.40E-05	4.36E-03	rs7957424	8.46E-06
7	*DDC*	372	1000000	50493627	50600648	5.70E-05	4.48E-03	rs4245556	9.88E-07
14	*COQ6*	130	1000000	73486395	73499566	5.80E-05	4.52E-03	rs7157715	9.25E-06
12	*GATC*	81	1000000	119368666	119382145	5.90E-05	4.56E-03	rs7957424	8.46E-06
11	*C11orf80*	61	1000000	66268782	66367563	6.30E-05	4.72E-03	rs10896139	1.78E-05
22	*FAM83F*	112	1000000	38720898	38755989	6.40E-05	4.76E-03	rs3021269	5.27E-05
19	*ZNF473*	230	1000000	55221023	55243843	6.40E-05	4.76E-03	rs11883067	5.09E-03
11	*LRFN4*	46	1000000	66381451	66384522	6.70E-05	4.87E-03	rs10896139	1.78E-05
14	*FAM161B*	140	1000000	73470457	73486588	7.00E-05	4.99E-03	rs7157715	9.25E-06
11	*RCE1*	39	1000000	66367458	66370579	7.10E-05	5.02E-03	rs10896139	1.78E-05
15	*PARP6*	121	1000000	70320575	70350682	7.10E-05	5.02E-03	rs1037680	7.21E-05
22	*TCF20*	140	1000000	40885962	40941389	7.30E-05	5.10E-03	rs5751229	1.08E-05
17	*AOC2*	46	1000000	38250134	38256251	7.30E-05	5.10E-03	rs9913866	3.66E-02
2	*GALNT5*	153	1000000	157822585	157876159	7.70E-05	5.24E-03	rs6437054	2.26E-05
2	*NPAS2*	424	1000000	100803044	100979719	8.10E-05	5.38E-03	rs6747755	5.97E-05
1	*COL9A2*	88	1000000	40538749	40555526	8.20E-05	5.42E-03	rs209923	5.28E-04
6	*COL11A2*	329	1000000	33238446	33268223	8.30E-05	5.45E-03	rs3117027	4.46E-06
2	*ILKAP*	113	1000000	238743781	238777063	8.30E-05	5.45E-03	rs7595215	6.13E-04
1	*TXNDC12*	112	1000000	52258391	52293635	8.60E-05	5.55E-03	rs12047841	5.19E-03
12	*PA2G4*	69	1000000	54784369	54793961	8.70E-05	5.59E-03	rs773123	2.26E-04
4	*BDH2*	92	1000000	104218230	104240473	9.10E-05	5.72E-03	rs11728063	2.13E-06
7	*POLR2J*	41	1000000	101900552	101906386	9.10E-05	5.72E-03	rs3823656	3.26E-04
14	*BEGAIN*	112	1000000	100073240	100105884	9.20E-05	5.75E-03	rs10148448	7.69E-05
9	*PPP2R4*	343	1000000	130913064	130951044	9.30E-05	5.78E-03	rs3118639	7.07E-05
13	*FBXL3*	120	1000000	76477396	76499258	9.40E-05	5.82E-03	rs2106227	1.39E-06
11	*LPXN*	144	1000000	58050919	58099910	9.50E-05	5.85E-03	rs2275993	8.07E-05
12	*ERBB3*	76	1000000	54760158	54783395	9.60E-05	5.88E-03	rs773123	2.26E-04
9	*DOLPP1*	215	1000000	130883226	130892538	9.80E-05	5.94E-03	rs3118639	7.07E-05

Supplementary Table 12. Gene-set and tissue enrichment analysis of continuous chronotype

Original gene set ID	Original gene set description	Nominal P value	False discovery rate
GO:0042596	fear response	1.00E-05	<0.20
GO:0002209	behavioral defense response	2.42E-05	<0.20
GO:0021795	cerebral cortex cell migration	1.66E-04	>=0.20
GO:0001662	behavioral fear response	1.77E-04	>=0.20
GO:0021987	cerebral cortex development	2.54E-04	>=0.20
ENSG00000156052	GNAQ PPI subnetwork	2.69E-04	>=0.20
GO:0033555	multicellular organismal response to stress	3.23E-04	>=0.20
ENSG00000156049	GNA14 PPI subnetwork	4.59E-04	>=0.20
GO:0031646	positive regulation of neurological system process	6.47E-04	>=0.20
MP:0011085	complete postnatal lethality	6.63E-04	>=0.20
GO:0010165	response to X-ray	7.31E-04	>=0.20
GO:0021952	central nervous system projection neuron axonogenesis	7.76E-04	>=0.20
ENSG00000162772	ATF3 PPI subnetwork	8.21E-04	>=0.20
REACTOME_G_ALPHA_Q_SIGNALLING_EVENTS	REACTOME_G_ALPHA_Q_SIGNALLING_EVENTS	9.17E-04	>=0.20
ENSG00000060558	GNA15 PPI subnetwork	9.34E-04	>=0.20

Name	MeSH first level term: MeSH second level term	Nominal P value	False discovery rate
Basal Ganglia	Nervous System: Central Nervous System	0.02	>=0.20
Corpus Striatum	Nervous System: Central Nervous System	0.03	>=0.20
Neutrophils	Hemic and Immune Systems: Immune System	0.05	>=0.20
Leukocytes	Cells: Blood Cells	0.05	>=0.20
Granulocytes	Cells: Blood Cells	0.05	>=0.20

Supplementary Table 13. Risk score for chronotype is causally associated with educational attainment.

Risk score for educational attainment, schizophrenia, and BMI are not associated with chronotype.

	**Beta**	**SE**	***P***	**SNPs**
Educational Attainment Risk Score (3 SNPs)	0.00265	0.00275	0.33481	3
Schizophrenia Risk Score (92 SNPs)	0.00077	0.00055	0.16271	92
BMI Risk Score (93 SNPs)	-0.00094	0.00052	0.07044	93

Risk scores were comprised of genome-wide significant SNPs from previous large-scale studies, weighted by the effect estimate.

Risk score (15 SNPs) for evening chronotype is associated with educational attainment

**Outcome**	**Beta**	**SE**	***P***
BMI	-3.71E-04	3.47E-04	0.2853
Schizophrenia	6.76E-05	4.11E-05	0.1005
Educational Attainment	8.53E-03	3.57E-03	**0.0167**

Chronotype risk score was comprised of all SNPs reported as GWS by 23andMe, and tested against phenotypes reported in the UKBiobank.

Mendelian Randomization of chronotype (as instrumented by the risk score) on Educational Attainment

	**Beta**	**SE**	***P***	**N**
chronotype (instrumented by risk score)	0.62539	2.70E-01	**0.02067**	68,718

Mendelian Randomization was performed using TSLS in R SEM package.

Supplementary Note 1. Candidate Chronotype Genes under the association peaks.

Candidate Chronotype Genes

MCL1 *(*1q21)- encodes an anti-apoptotic protein, which is a member of the Bcl-2 family. *MCL1* mRNA is rhythmically expressed in the liver. Knock-down of *MCL1* results in disruption of circadian rhythms and the promoter of *MCL1* is bound by known circadian transcription factors.

ZZZ3 *(*1p31.1)-component of the ATAC complex. Contains binding sites for several known circadian transcription factors, is linked to GWAS for obesity, and knock-down disrupts circadian rhythms.

RPRD2 *(*1q21.3)-interacts with the RNA polymerase II complex. In a yeast-2-hybrid screen, *RPRD2* binds *PPP1CA,* a known regulator of circadian rhythms. *RPRD2* is rhythmically expressed in the suprachiasmatic nucleus, the central pacemaker. Knock-down of *RPRD2* alters circadian rhythms.

TNRC6B *(*22q13.1)-plays a role in RNA-mediated gene silencing. *TNRC6B* controls circadian behavior in flies and is bound by known circadian transcription factors

POLR2J *(*7q22.1)-a subunit of RNA polymerase II. Knock-down of *POLR2J* results in disruption of circadian rhythms and the promoter of *POLR2J* is bound by known circadian transcription factors

ERC2 *(*3p14.3)-thought to be involved in the regulation of neurotransmitter release. The fly ortholog is expressed rhythmically, influenced by light, and is involved in circadian plasticity in the visual system.

HTR6 *(*1p36-p35)-receptor is thought to regulate cholinergic neuronal transmission in the brain. *HTR6* is known to regulate the sleep wake cycle and, in flies, loss-of-function results in decreased sleep wake arousal and rescue of a short sleep phenotype caused by mutations in the dopamine transporter.

Candidate genes with known role in circadian rhythms

PER2 *(*2q37.3)-part of the transcriptional/translational feedback loop of the core circadian clock. *PER2* is rhythmically expressed in the suprachiasmatic nucleus, the central pacemaker. *Per2* knock-out mice have a short period and some *Per2* knock-out mice are arrythmic in constant darkness.

APH1A *(*1p36.13-q31.3)- encodes a component of the gamma secretase complex. *APH1A* interacts with *ATF4* a known regulator of circadian transcription. It is also rhythmically expressed and bound by known circadian transcription factors. *APH1A* is also reported to be involved in the “sundown” effect seen in Alzheimer’s patients.

RGS16 *(*1q25-q31)-belongs to the 'regulator of G protein signaling' family. Deficiency of *RGS16* lengthens the period of activity in mice. Knock-out also leads to attenuated food anticipatory activity. *RGS16* is diurnally regulated in the liver and is responsible for a dis-inhibition of cAMP synthesis that starts the circadian rhythm of the suprachiasmatic nucleus.

FBXL13 *(*7q22.1)-acts as a protein-ubiquitin ligase. Core circadian rhythm proteins, CRY1/2, are degraded in a process dependent on *FBXL13.* Fbxl3 murine mutants have a slower clock.
